# Peptidomics and pharmacological profiling of *Odontobuthus doriae* (Buthidae) scorpion venom at the kappa opioid receptor

**DOI:** 10.1038/s41598-025-31108-9

**Published:** 2026-01-31

**Authors:** Adel Abdollahnia, Boglarka Blanka Bata, Andreas Fraunhofer, Julius Hermes, Javad Atashi, Alireza Ghassempour, Christian W. Gruber

**Affiliations:** 1 Institute of Pharmacology, Center for Physiology and Pharmacology, Medical University of Vienna, Vienna, Austria; 2 Medicinal Plants and Drugs Research Institute, Shahid Beheshti University, G.C. Evin, Tehran, Iran

**Keywords:** scorpion venom, peptide toxin, MALDI-TOF, mass spectrometry, Biochemistry, Chemical biology, Chemistry, Drug discovery

## Abstract

Scorpion venoms are rich in bioactive peptides, many of which act on ion channels and neurotransmitter systems, yet their capacity to interact with G protein-coupled receptors (GPCRs) has been largely unexplored. Here, we profiled the venom peptides of five species in the family Buthidae and evaluated their activity at the kappa opioid receptor (KOR). Matrix-assisted laser desorption/ionization time-of-flight mass spectrometry (MALDI-TOF MS) revealed species-specific peptide fingerprints in the mass range between 2.5 and 4 kDa, underscoring interspecies peptide toxin variation. Following pre-purification by solid-phase extraction, radioligand displacement assays demonstrated that fractions from *Odontobuthus doriae* bound to KOR, with Od-e (36% acetonitrile) and Od-f (45% acetonitrile) displacing ~ 35–40% of [³H]-diprenorphine. However, BRET-based functional assays demonstrated only weak receptor activation, suggesting that these peptides may function as partial agonists or antagonists rather than full agonists. Collectively, these findings highlight scorpion venoms as a previously underexplored source of opioid receptor-interacting peptides. Systematic investigation of their structural diversity and pharmacological profiles in the future may not only expand our understanding of venom evolution but also provide novel scaffolds for GPCR ligand discovery and potential analgesic development.

**Supplementary Information:**

The online version contains supplementary material available at 10.1038/s41598-025-31108-9.

## Introduction

Venoms are a rich source for discovering bioactive compounds^1^. Among venom-producing animals, snakes, scorpions, and spiders represent an especially important reservoir of peptides and proteins with diverse and potent biological activity^2^. Evolutionary divergence across families, genera, and habitats has produced wide variation in venom composition, resulting in remarkable structural and molecular diversity^3–5^. Venoms serve ecological functions such as prey immobilization, defense, and intraspecific competition^6^, and their major constituents include proteins, peptides, lipids, and enzymes^7^.

Scorpions are the most widely distributed venomous species, with approximately 2772 species identified, including around 104 of medical importance^8^. Members of the Buthidae family are responsible for many scorpion stings worldwide^9^, making these inicidents a majorl health concern. Scorpion venoms are biochemically complex, containing diverse bioactive molecules^10^. Previous analytical studies using chromatography and mass spectrometry have revealed the presence of peptides^11,12^, with sodium- and potassium-channel inhibitors being the most common^13^.

Recent work has identified several venom-derived peptides with analgesic potential. Ion channel blockers from scorpions have been highlighted as non-opioid analgesic candidates. For example, Leptucin, a cysteine-rich 55-amino acid peptide isolated from *Hemiscorpius lepturus*, produced strong analgesic effects in animal models^14^. Buthicyclin, a cyclic peptide inspired by a defensin from *Mesobuthus martensii*, showed potent, long-lasting pain relief mediated through mu- (MOR) and kappa- (KOR) opioid receptors^15^. Similarly, BmK-YA, a peptide from the same species and structurally related to endogenous enkephalins, acts as a partial agonist at the MOR and displays promising analgesic properties^16^. These findings highlight scorpion venoms, particularly from Buthidae, as valuable sources for identifying peptides with activity at the opioid system^17^.

The endogenous opioid system, consisting of MOR, KOR and delta-opioid receptors, plays a central role in pain modulation^18^. MOR activation produces strong analgesia, but carries high addiction risks, whereas KOR activation is less prone to abuse but often associated with dysphoria^19^. The pharmacological potential of KOR in pain management remains an open research question, particularly regarding the discovery of novel peptide ligands from natural sources^20^. Beyond their therapeutic promise, such ligands are urgently needed as molecular tools to probe opioid receptor function and as leads for drug discovery^21^.

Building on this rationale, the present study investigated venoms from five Iranian scorpion species, i.e. *Androctonus crassicauda*,* Odontobuthus doriae*,* Mesobuthus eupeus*,* Hottentotta zagrosensis*, and *Hottentotta saulcyi*, all members of the Buthidae family. The objective of this research was to evaluate the effects of venom extracts as modulator of KOR signaling. Crude venoms and semi-purified fractions were analyzed, and peptide components were characterized through separation via solid-phase extraction (SPE) and peptidomics. By combining matrix-assisted laser desorption/ionization time-of-flight mass spectrometry (MALDI-TOF MS) with transcriptomic data, including the analysis of open reading frames and their putative post-translational modifications, we established a rapid method for systematic identification and characterization of venom peptides with potential KOR activity.

## Methods and materials

### Solvents and reagents

Materials, reagents, and solvents required were: [^3^H]-diprenorphine (DPN) and jetPRIME transfection reagent from (Revvity, USA), as well as other materials including 2-(3,4-Dichlorophenyl)-N-methyl-N-[(1R,2R)−2-(1-pyrrolidinyl)cyclohexyl]acetamide(U50,488 H), Magnesium chloride hexahydrate (MgCl_2_), bovine serum albumin (BSA), EDTA. The α−4-hydroxycinnamic acid matrix (CHCA), trifluoroacetic acid (TFA), methanol (MeOH), acetonitrile (ACN) and polyethylenimine, all with HPLC-grade purity were purchased from (Sigma-Aldrich, Germany).

### Preparation of venom samples

#### Collecting scorpion species

All scorpion species required for this study, including *A. crassicauda* (Ac), *O. doriae* (Od), *M. eupeus* (Me), *H. saulcyi* (Hs), and *H. zagrosensis* (Hz) from the Butidae family, were obtained from a scorpion breeding farm in Larestan, Fars Province, Iran. Adult scorpions (two- to four years old; equal ratio of both sexes) were maintained on a diet of *Tenebrio molitor* (mealworms) and venom samples were collected and pooled for experimentation. To stabilize venom composition and minimize physiological variation, a five-day fasting period was empirically implemented before venom extraction. The number of individual animals per species varied between 1,500 and 4,000 (depending on body size). Venom extraction was conducted during the summer season (in June).

#### Venom extraction

Crude venom was obtained by electrically stimulating the scorpions’ telsons using an electro-pulse stimulator with a voltage between 5 and 10 volts. The extracted venom, which had a milky white appearance, was transferred to sterile vials and stored in a nitrogen tank until transported to the laboratory. The collected venom samples were first lyophilized for 48 h using a freeze dryer model BETA 2–8 LSCplus (Martin Christ, Germany) and then stored at −20 °C for subsequent analysis in aliquots. The average yield of dried venom per species was approximately 200 mg.

### Venom sample preparation and pre-purification

The analysis started with dissolving 60 mg of crude venom in 60 mL of 5% solvent B (90% ACN, 0.1% TFA) in 95% solvent A (H_2_O, 0.1% TFA). After 2 min of vortexing and centrifugation at 3500 × g at 4 °C for 15 min, the mixture was poured through a 0.22 μm syringe filter. SPE cartridges C18-E (1 g, 55 μm, 70 Å; Phenomenex, Germany) were used for fractionation. Following activation with MeOH and equilibration with solvent A, venom peptides were stepwise eluted with 20, 30, 40, 50, 60, 80, 90 and 100% of solvent B. The fractions were collected, dried, freeze-dried, and stored at − 20 °C for further analysis. Next, analytical reverse-phase high-performance liquid chromatography (RP-HPLC) was performed using a DIONEX UltiMate 3000 UHPLC system (Thermo Scientific, Austria). Separation was performed on a Kinetex C18 column (150 mm × 3 mm, 2.6 μm, 100 Å; Phenomenex) at a constant flow rate of 0.4 mL/min. Peptide elution was performed applying a linear gradient of 2%/min solvent B from 5 to 65%. Elution of peaks was monitored by measuring ultraviolet (UV) absorption at a wavelength of 214 nm.

### Mass spectrometry and identification of venom components

MALDI-TOF MS analysis of crude scorpion venom and SPE fractions was performed on an autoflex MALDI-TOF/TOF analyzer (Bruker Daltonics, Germany). MS spectra were recorded in positive ion reflection mode, and laser intensity was optimized for each spectrum (2000 shots per spot). Samples were prepared by mixing 3 µL of CHCA matrix and 0.5 µL of peptide and applying 0.5 µL of the mixture onto a MALDI 384 target plate. Spectra were acquired, processed, and analyzed using Flex Analysis 3.4 software (Bruker Daltonics).

### Cell culture and membrane preparation

As described previously^22^, HEK293 (ATCC CRL-1573) or HEK293T (ATCC CRL-3216) cells were cultured at 37 °C in a humidified incubator with 5% CO₂ using Dulbecco’s Modified Eagle Medium (DMEM, high glucose; Gibco, USA), supplemented with 10% fetal bovine serum (FBS; Gibco, USA), 50 U/mL penicillin, and 50 µg/mL streptomycin to support cell growth and prevent bacterial contamination. HEK293 cells stably expressing the murine KOR (mKOR) were cultured with the addition of 0.8 mg/mL geneticin (G418; Thermo Fisher Scientific, USA) for selection. At around 90% confluency, cells were harvested on ice by scraping, then pelleted by centrifugation at 3,000 × g for 5 min at 4 °C and resuspended in 1 mL HME buffer (50 mM Tris, 5 mM MgCl_2_, 0.1% BSA) with Complete protease inhibitor cocktail (Roche, Germany). Cell membranes were homogenized, washed and isolated by centrifugation at 20,000 rpm at 4 °C for 20 min. The resulting pellet was resuspended in HME buffer and protein concentration was determined using a BCA kit and BSA as the quantitative standard protein (Sigma-Aldrich, Germany). Membranes were stored at −80 °C until further use.

### Radioligand binding assay

The membranes used for the radioligand displacement binding assay were prepared from stably transfected cells expressing the mKOR and quantified as described previously^23^. Radioligand displacement binding assays were carried out in a final volume of 300 µL. Each assay mixture containing [^3^H]-diprenorphine (3 nM) radioligand, HME buffer supplemented with 1 mg/mL bovine serum albumin (BSA), competing unlabeled ligands, crude venom extracts or fractions and membranes from stable cells expressing mKOR (4 µg/assay). Non-specific binding was determined in the presence of 1 µM naloxone; U50,488 and dynorphin A_1 − 13_ (1 µM each) were used as positive controls. Competing crude venom and fractions were prepared at 4x concentration, with final concentrations of 30 µg/mL and 100 µg/mL in the assay. All samples were incubated at 37 °C for 1 h followed by filtration to a 0.1% polyethlyenimine-soaked GF/C glass fiber filter (Sartorius, Germany) by a Skatron cell harvester (Skatron AS, Norway). Filters containing receptor-bound radioligand were transferred into scintillation vials and incubated with 2 mL Rotiszint scintillation cocktail (Carl Roth, Germany) in each tube for 1 h while shaking. Radioactivity, expressed as counts per minute was quantified by liquid scintillation using a Beckman Coulter LS6500 (Beckman Coulter Inc, USA). Each experimental condition was performed in technical duplicates, and in two independent measurements (*n* = 2).

### G protein dissociation-based BRET assay

G protein dissociation was measured in a Bioluminescence Resonance Energy Transfer (BRET) assay. To measure G protein activation, wild type HEK293T cells were maintained in a 10 cm cell culture dish in DMEM containing phenol red and L-glutamine, supplemented with 10% FBS and 1% penicillin/streptomycin (P/S). Cells were split into 6-well plates and grown under standard culture conditions (37 °C, 5% CO₂) until reaching 70–80% confluency, then they were transiently transfected with 2000 ng plasmid DNA containing the human KOR gene OPRK1, 2000 ng Gα-nLuc and Gßγ-cpVenus tagged G protein plasmid (as described by Schihada et al.)^24^ and 8 µl jetPRIME transfection reagent (Polyplus, France) in 200 µl jetPRIME transfection buffer. The cell transfection was performed according to the manufacturer’s protocol. The transfected cells were incubated for 4–6 h, seeded into white clear-bottom 96-well cell culture plates in 100 µL DMEM phenol red-free media supplemented with 10% FBS and 1% P/S at a cell density of 50,000 cells/100 µL. The 96-well plates were incubated overnight at 37 °C and 5% CO_2_.1 h prior to measurement, the cells were starved in 100 µL DMEM phenol red-free media lacking supplemented FBS and P/S. Ligands and 1:100 diluted nLuc substrate (e.g. furimazine or hikarazine) were prepared in Hank’s Balanced Salt Solution (HBSS). U50,488 and dynorphin A_1 − 13_ (final concentration 1 µM) were used as positive controls. Scorpion peptide fractions were tested at final concentrations of 30 µg/mL and 100 µg/mL. 50 µL of diluted luciferase reagent was added to each well and incubated for 5 min at 37 °C. 5 min after starting the measurement, 50 µL of the ligands were added by the FlexStation 3 plate reader, then BRET ratios were recorded for 20 min. Single concentration flex measurements were performed in technical duplicates. In total, each well contained 100 µL DMEM medium, 50 µL 1:100 diluted luciferase substrate, 50 µL of the ligand solution or 50 µL HBSS (used the establish a baseline). Emissions were measured simultaneously at 460 nm (donor, nLuc) and 530 nm (acceptor, cpVenus). The final assay volume per well was 200 µL (100 µL DMEM, 50 µL diluted assay reagent, and 50 µL of ligand). Ligand induced G protein activation was shown as the decrease of the BRET ratio. Each condition was measured in technical duplicates in two separate measurements (*n* = 2).

### Data analysis

GraphPad Prism software (version 8.4.3) was used to analyze ligand binding data, including normalization and correction for non-specific binding. For protein and peptide identification, mass spectrometry data were further processed by searching against publicly available databases. Protein and peptide sequences from the scorpion family Buthidae (taxonomic ID: 6855) and the species *O. doriae* (taxonomic ID: 342590) were retrieved from UniProt (https://www.uniprot.org, accessed July 12, 2025) and NCBI (https://www.ncbi.nlm.nih.gov, accessed July 12, 2025) databases. To ensure accurate identification of venom-derived proteins and peptides, the searches were specifically limited to the taxonomic classifications of these species. The peptide fragmentation and post translational modification analysis was performed using an in-house script.

## Results

### MALDI-TOF mass spectrometry analysis of crude Scorpion venom

**Fig. 1:**
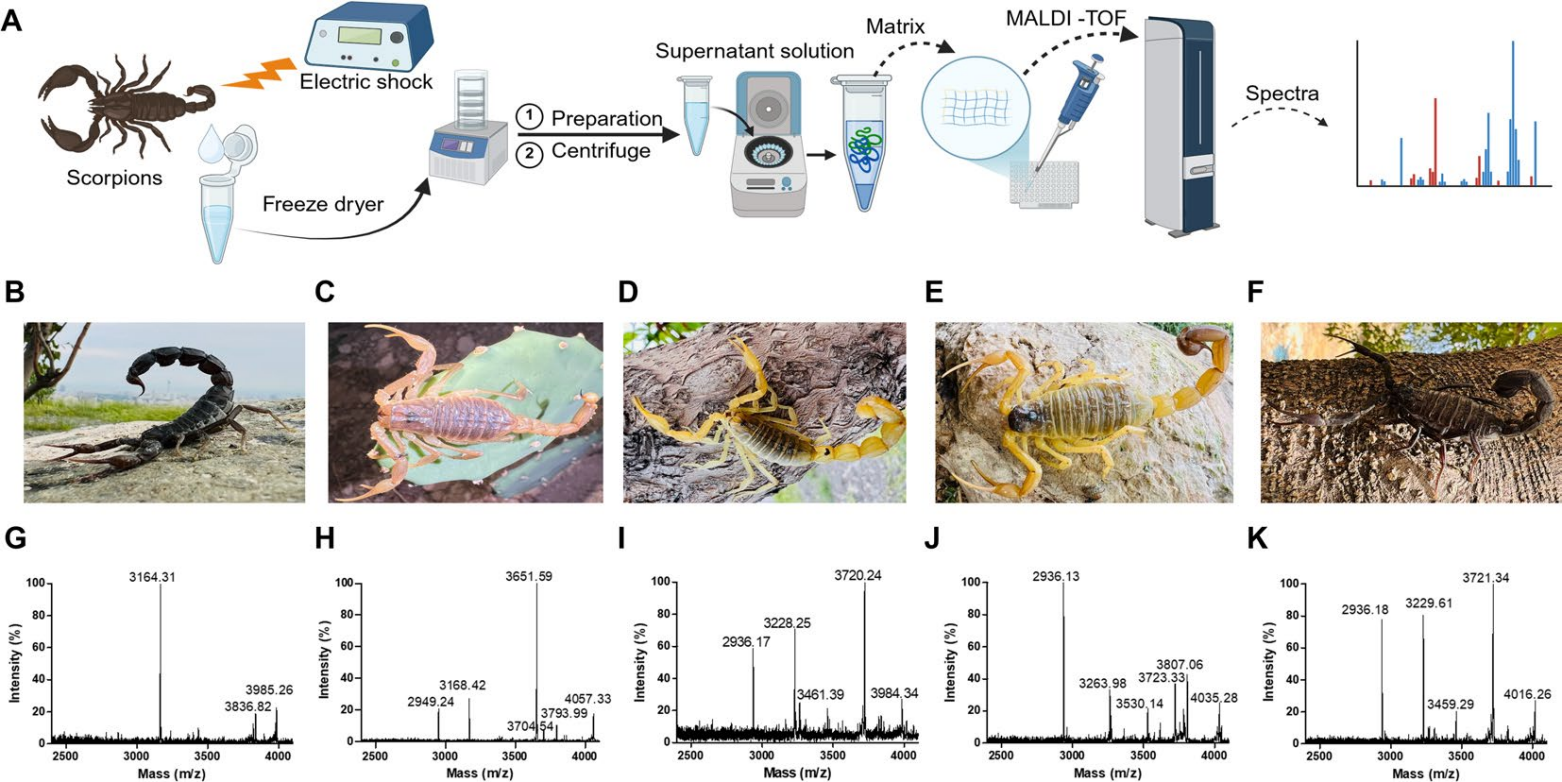
Scorpion venom peptide analysis. Overall workflow is presented in (**A**). Images of scorpion species, i.e. *A. crassicauda* (**B**), *O. doriae* (**C**), *M. eupeus* (**D**), *H. saulcyi* (**E**), and *H. zagrosensis* (**F**). The photographs were captured by Javad Atashi. (**G**-**K**) MALDI-TOF MS analysis of crude venom samples from the five scorpion species (same order).

First, the crude venom extracts of five scorpion species were analyzed by MALDI-TOF MS, to identify possible peptide peaks. Scorpion venom typically contains peptides from ~ 1–8 kDa^25^. Since we previously identified KOR-active peptides from plants with masses of ~ 3 kDa^26^, we focused our analysis here on the m/z 2500–4000 range. A schematic of the sample preparation and analytical workflow is shown in Fig. 1A, outlining the key experimental steps. To facilitate visual identification, photographs of the scorpions in their natural habitats, highlighting differences in body size and coloration, are provided in Fig. 1B–F. Representative MALDI-TOF MS spectra for each species are shown in Fig. 1G–K, recorded in the molecular weight range from, i.e. the mass range where most peptides were detected (m/z 2500–4100). As anticipated, some species exhibited distinguishable peptide fingerprints, whereas others showed overlapping yet comparable profiles. Given that each venom sample represented a pool of up to 4,000 individuals of mixed sex, these profiles should be regarded as averaged representations rather than individual fingerprints. Nevertheless, differences in peak distribution and relative signal intensity provide a valuable basis for interspecies differentiation. There were three to six dominant peaks (> 10% base peak intensity) identified per species (Fig. 1G–K). A characteristic signal at m/z ~ 2900 was absent in *A. crassicauda* but present in the other species, with particularly high intensity in *H. zagrosensis*. These spectral differences demonstrate the value of MALDI-TOF MS as a reliable and rapid method for differentiating scorpion species based on venom peptide profiles. The complete list of molecular masses (m/z) for each scorpion species are provided in Supplementary Information Table S1 and Fig. S1.

### Purification, chromatography and mass spectrometry of venom peptide fractions

**Fig. 2:**
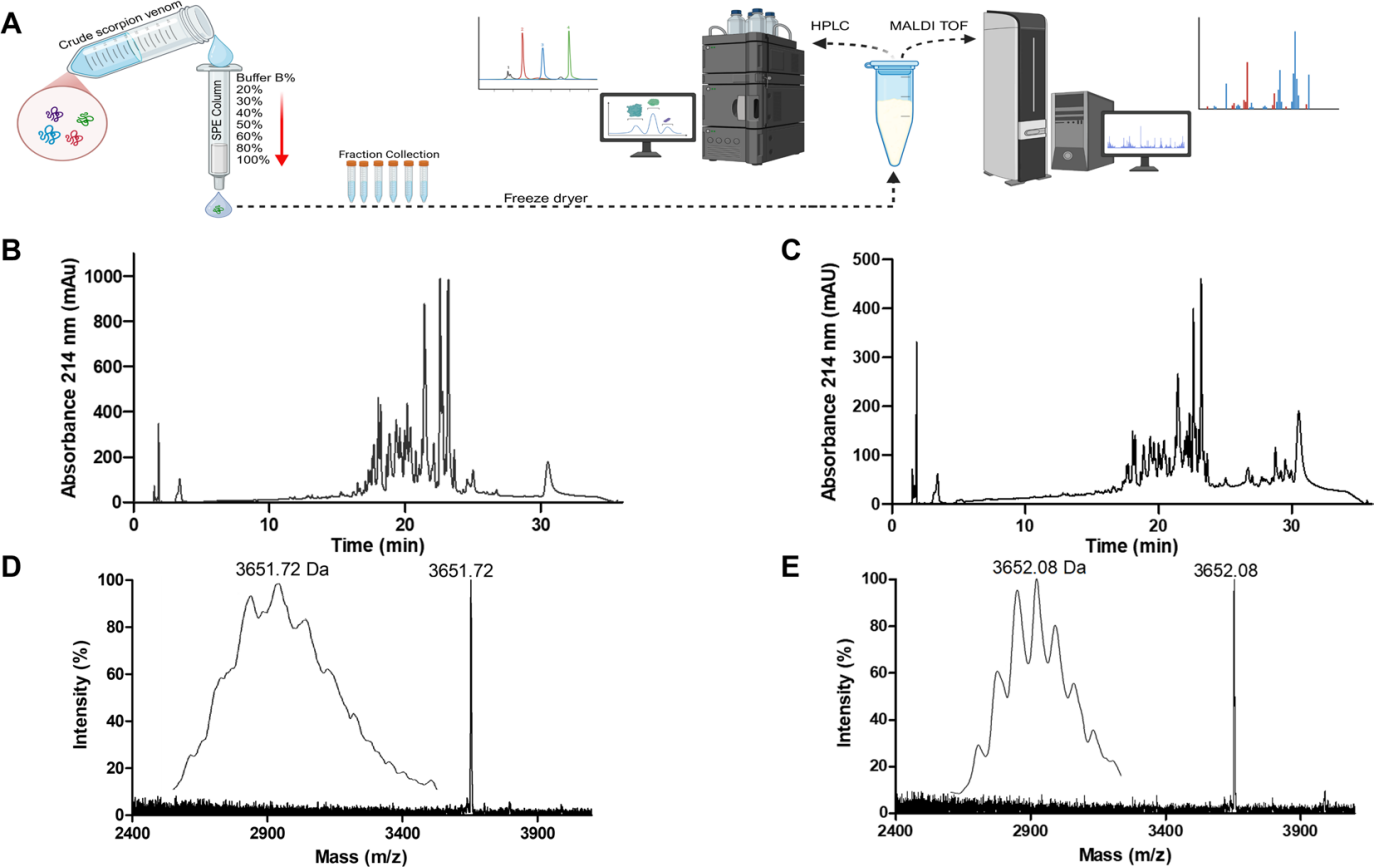
Preparation and analysis of venom peptide fractions. (**A**) Workflow representation: crude venom samples were pre-purified using solid-phase extraction (SPE) and further analyzed by HPLC and MALDI-TOF MS. HPLC chromatograms of the fractions Od-e (**B**) and Od-f (**C**) are shown. MALDI-TOF MS analysis of the venom fractions in the mass range of m/z 2500–4000, of fractions Od-e (**D**) and Od-f (**E**) with the average with the average mass peaks ([M + H]^+^) labelled The zoomed inset to the left of each spectra shows the monoisotopic peaks.

Scorpion venom fractions were prepared and analyzed using a three-step workflow involving purification, HPLC, and mass spectrometry (Fig. 2). Crude venom samples were subjected to SPE to remove salts and other interfering substances, yielding semi-purified fractions for further analysis (Fig. 2A). This purification step improved the reproducibility and accuracy of subsequent chromatographic and mass spectrometric measurements. Fractions were then collected according to different gradients of solvent B, and their activity guided further analysis. Exemplarily, for *O. doriae*, two fractions, i.e. Od-e (40% solvent B, i.e. 36% ACN) and Od-f (50% solvent B, ~ 45% ACN) were analyzed, each showing a large number of peaks with varying intensities (Fig. 2B, C). Notably, the fraction Od-f displayed a higher number of peaks with better resolution in the retention time range of 25 to 33 min. MALDI-TOF spectra of the fractions revealed distinct peaks with small mass differences, consistent with the presence of closely related molecular components. A prominent signal at m/z 3654.04 ([M + H]^+^, average mass) was identified in Od-e, while a closely related peak at m/z 3654.00 ([M + H]^+^, average mass) was observed in Od-f, indicating species-specific signals (Fig. 2D, E).

### Comparative analysis of scorpion venoms and peptide fractions targeting KOR

**Fig. 3:**
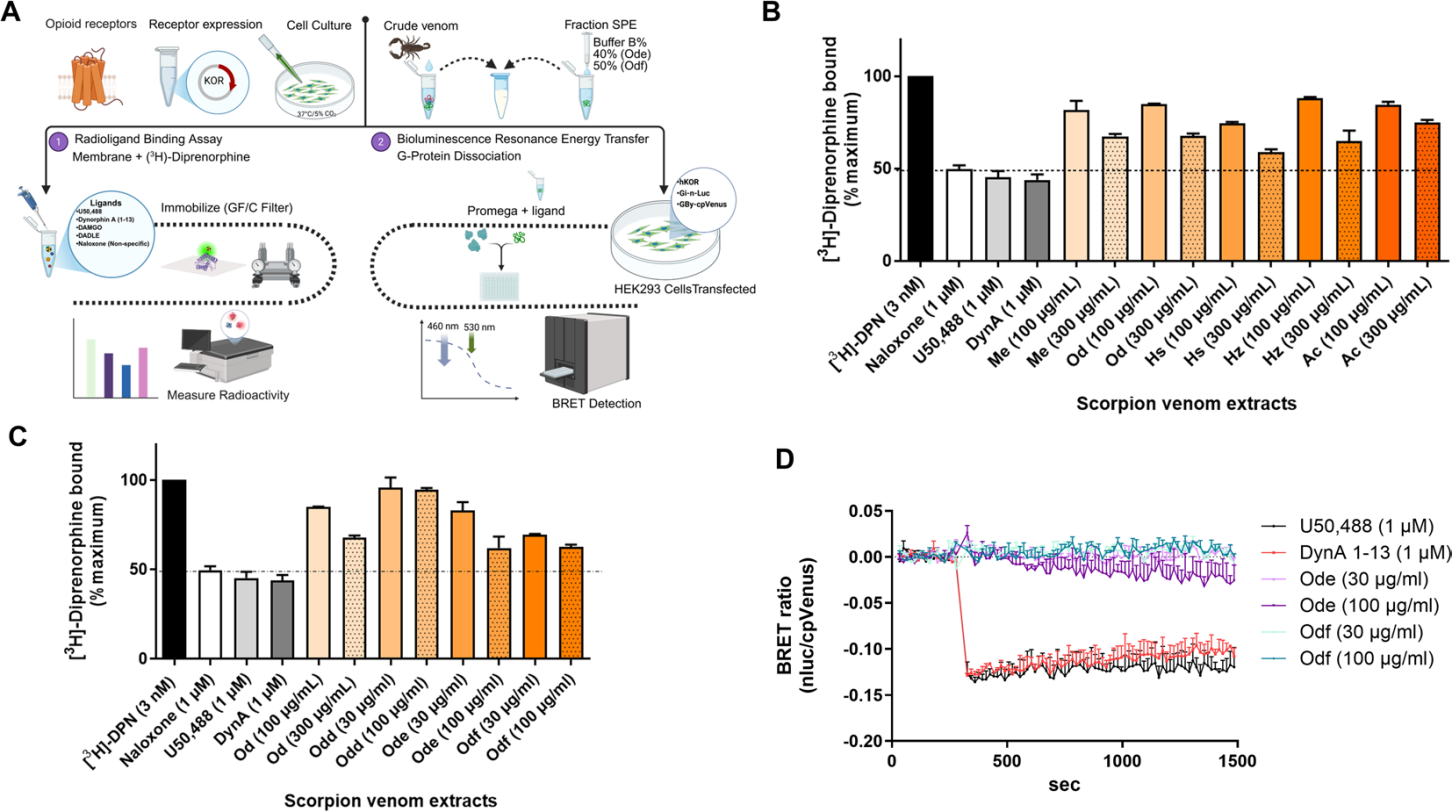
Pharmacological profiling of scorpion venom peptide fractions at KOR. (**A**) Schematic representation of the experimental workflow, including binding assays to opioid receptor (KOR) and G protein BRET analysis. (**B**) Binding of crude venom extracts from Ac, Od, Me, Hs, and Hz for the KOR (*n* = 2). (**C**) Comparative analysis of SPE-purified venom fractions from the Od species, with emphasis on the effect of purification on receptor binding patterns (*n* = 2), (D) Functional activity of fractions Od-e and Od-f; G protein activation at KOR was assessed via BRET and no response was observed for any of the fractions (*n* = 2).

To investigate the pharmacological properties of scorpion venoms at KOR, the experimental workflow, including receptor binding assays and G protein biosensor analysis, is summarized in Fig. 3A. As a first step, crude venom extracts from *A. crassicauda* (Ac), *O. doriae* (Od), *M. eupeus* (Me), *H. zagrosensis* (Hz), and *H. saulcyi* (Hs) were tested for KOR binding at concentrations of 100 and 300 µg/mL. As shown in Fig. 3B and Supplementary Table S2, the Hs crude extract exhibited on average approximately 16% higher radioligand displacement than Ac at the higher concentration (300 µg/mL), whereas Od, Me, and Hz venoms achieved around 35% displacement of radioligand. The binding of venom extracts remained weak compared to the reference ligands U50,488, naloxone, and dynorphin A_1 − 13_ at 1 µM. Purified fractions obtained by SPE under varying buffer gradients were then assessed. Among these, Od-e and Od-f (derived from *O. doriae*) showed stronger binding (radioligand displacement) at 100 µg/mL than fractions from other species (Supplementary Information Fig. **S2** and Table S2). As illustrated in Fig. 3C, both fractions Od-e (38% displacement) and Od-f (37%) bound stronger at 100 µg/mL as compared to Od-d (6%) and exhibited comparable radioligand displacement to crude Od venom (15%). The functional effects of Od-e and Od-f were tested in the G protein BRET-based biosensor assay (Fig. 3D). Dynorphin A_1 − 13_ produced a marked decrease in the BRET ratio, consistent with robust G protein activation, while U50,488 caused a similar but weaker response. In contrast, neither Od-e nor Od-f induced notable changes at 30–100 µg/mL, with signals remaining near baseline; however, Od-e displayed slightly stronger activity than Od-f at the higher concentration.

### In Silico fragmentation analysis of potential *Odontobuthus doriae* venom peptide precursors

**Fig. 4:**
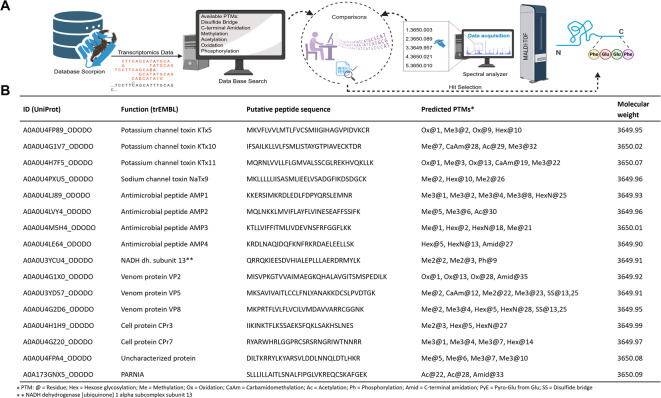
In silico analysis of the *O. doriae* peptidome. (**A**) Workflow for protein and peptide identification using MALDI-TOF MS combined with transcriptomics database searches (http://www.uniprot.org). (**B)** Putative sequence identification of peptides and proteins in *O. doriae* venom. The table summarizes the putative sequences of the active fractions Od-e and Od-f, identified from the components of *O. doriae* venom through transcriptomic and proteomic data analyses. Each entry is annotated with the UniProt ID, peptide/protein name, deduced amino acid sequence, possible post-translational modifications (PTMs), and the calculated molecular weight.

MALDI-TOF MS analysis of the Od fractions revealed distinct peptide signals with well-defined molecular masses. These spectra provided reproducible mass profiles for the active fractions, highlighting specific molecular components associated with the samples. Since the most prominent peaks of both fractions were observed at m/z 3650 (monoisotopic), we decided to do a basic in silico fragmentation analysis of possible precursor sequences for peptides of that mass, using an in-house script. The overall workflow is depicted in Fig. 4A. The analysis was performed on all protein sequences currently available for *O. doriae* in the trEMBL database. The sequences were partially fragmented on a set of potential protease cleavage sites (C-terminal side after K or R), and fitted with combinations of up to 6 post-translational modifications (PTM), including (poly-)methylation (Me) at arginine or lysine, oxidation (Ox) of methionine or cysteine, lysine acetylation (Ac), cysteine carbamidomethylation (CaAm), disulfide bridges (SS), C-terminal amidation (Amid), N-terminal pyroglutamation (PyE, PyQ, Glu or Gln) phosphorylation (Ph) and hexose glycosylation (Hex) on respective sites. All (modified) fragments within a ± 0.1 range of 3650 Da are reported. A subset of fragments matching the detected mass value is shown in Fig. 4B. The identified fragments ranged from 26 to 33 amino acids in length. Precursors included enzymes (e.g., NADH dehydrogenase 1 alpha subcomplex subunit 13), ion channel toxins (e.g., potassium channel toxins KTx5, KTx10 and KTx11, sodium channel toxin NaTx9), venom proteins (e.g., VP8), as well as antimicrobial peptides (AMP1, AMP2, AMP3, AMP4). In addition, cell-associated proteins (CPρ3, CPρ7) and an uncharacterized protein were included. Interestingly, the potassium and sodium channel toxin fragments (KTx and NaTx families) in the range showed multiple methylation and glycosylation sites, which may influence their stability and interaction with ion channels. The antimicrobial peptide originating fragments (AMP1–AMP4) exhibited combinations of methylation, glycosylation, and amidation, which are typical modifications enhancing antimicrobial potency and resistance to proteolytic degradation. Additional precursor details, as well as the full list of identified fragments can be found in Supplementary Data.

## Discussion

In this study, we demonstrate that scorpion venom extracts contain peptides capable of binding to the KOR, although functional responses in cellular assays were weak. This is noteworthy considering the previous discovery of BmK-YA, an enkephalin-like peptide from *M. martensii*, which has been identified as one of the most important opioid-related peptides from scorpion venom, acting as a potent agonist at the delta opioid receptor with weaker activity at MOR and KOR^16^.

Our findings therefore extend this line of evidence by demonstrating that other Buthidae venom preparations, including those from *O. doriae*, *Androctonus mauretanicus*^27^, *Hormurus waigiensis*^28^, *Androctonus australis*^13^, and *Hemiscorpius lepturus*^29^ also contain peptide components that can interact with opioid receptors, though their pharmacological effects remain weaker compared to BmK-YA. A central element of our workflow was the use of pre-purification strategies to handle the biochemical complexity of crude venoms. Direct application of unfractionated samples resulted in poor chromatographic resolution and inconsistent pharmacological readouts, largely due to interference with low-molecular-weight components such as salts, sugars, and other non-peptidic molecules. By employing SPE followed by RP-HPLC separation, peptide purity and analytical reproducibility were markedly improved, which enabled both reliable peptidomics profiling and receptor binding assays. These findings emphasize that systematic pre-purification is indispensable when studying complex venom mixtures^30,31^.

The different morphological traits of scorpions, such as color, size, and body structure, are mirrored in their venom MS profiles. Our findings indicate that even within the same family, each scorpion species displays a distinct mass fingerprint compared to other members of the same family, and such variation is also observed under intraspecific conditions. In a recent study of *Androctonus amoreuxi* and * Buthacus stockmanni*, two scorpion species found in Morocco, MALDI-TOF MS-derived mass fingerprinting was established as a promising tool for taxonomic identification of scorpions^32^. These findings are consistent with previous reports demonstrating that venom proteome fingerprints obtained by MALDI-TOF MS provide a reliable means for differentiating scorpion venoms^33^.

To date, a direct link between *O. doriae* venom and opioid receptor activity has not been reported. Previous studies on this species have instead described effects on neurotransmitter systems, such as acetylcholine, noradrenaline, glutamate, and GABA^34^, as well as on ion channels, particularly sodium and potassium channels, through toxins including Od1^35^ Odk1^36^ and Odk2^37^. In contrast, *M. martensii* has already yielded opioid-related peptides such as BmK-YA^15^^[,16^ and further examples include TsNTxP from *Tityus serrulatus*^38^ and hetlaxin from *Heterometrus laoticus*^39^, both with reported antinociceptive activity. These findings collectively support the view that scorpion venoms contain diverse pharmacologically relevant peptides.

Opioid receptors are known to interact with a defined set of endogenous peptide ligands: dynorphins for KOR^40,41^, enkephalins for DOR^42^, β-endorphin and endomorphins for MOR^43^, and nociceptin for the NOP receptor^44^. Despite their diversity, these peptides share conserved structural features that define the opioid peptide family^45^. In this study, we observed that scorpion venom fractions were able to bind to KOR, although functional activity was weak. These findings suggest that scorpion-derived peptides may possess sequence or structural elements sufficient for receptor recognition, even if they lack the high potency or selectivity of classical opioid peptides. Such interactions underline the broader potential of venom-derived peptides as a source of pharmacologically relevant molecules, especially in the context of GPCR ligand discovery^46^, and warrant further investigation into their role in receptor modulation in the future.

## Conclusion

This study investigated the interactions of scorpion venoms with opioid receptors, with a focus on the kappa subtype as an alternative opioid target in pain modulation. Radioligand displacement assays demonstrated receptor binding, although functional activity was weak, suggesting the possible presence of antagonistic peptides. Elucidating the pharmacological mechanisms underlying these interactions will require more detailed studies in the future. Given the remarkable diversity of scorpion species and the complexity of their venoms, continued research is essential to define the molecular basis of receptor binding and evaluate its biological relevance. In this context, systematic exploration of scorpion venoms may provide valuable insights into peptide diversity and offer new perspectives on receptor modulation.

## Supplementary Information

Below is the link to the electronic supplementary material.

Supplementary Material 1

Supplementary Material 2

## Data Availability

Original and raw data, as well as scripts for transcriptome and peptide analysis are available upon reasonable requests from the corresponding authors.
