# Supplementary material for: Peptidomics and pharmacological profiling of Odontobuthus doriae (Buthidae) scorpion venom at the kappa opioid receptor
Source: Sci Rep. 2026 Jan 31;16:4536. doi: 10.1038/s41598-025-31108-9 (PMC12868723; doi:10.1038/s41598-025-31108-9)
Supplement: Supplementary file 1 — Supplementary Material 1 [file 41598_2025_31108_MOESM1_ESM.docx]

**Supplementary Information**

**Peptidomics and pharmacological profiling of *Odontobuthus doriae* (Buthidae) scorpion venom at the kappa opioid receptor**

Adel Abdollahnia^1,2^, Boglarka Blanka Bata^1^, Andreas Fraunhofer^1^, Julius Hermes^1^, Javad Atashi^2^, Alireza Ghassempour^2^ and Christian W. Gruber^1^

^1^Institute of Pharmacology, Center for Physiology and Pharmacology, Medical University of Vienna, Vienna, Austria

^2^Medicinal Plants and Drugs Research Institute, Shahid Beheshti University, G.C. Evin, Tehran, Iran

**Table of Contents**

| **Name** | **Description** |
| --- | --- |
| **Supplementary Figure S1** | Profile MALDI-TOF MS chromatograms of scorpion crude venoms from five studied species |
| **Supplementary Figure S2** | Binding (competitive displacement) of scorpion crude venom extracts and fractions at the KOR |
| **Supplementary Table S1** | MALDI-TOF MS profiles of crude venom from scorpion species |
| **Supplementary Table S2** | Normalized binding data of scorpion crude venom extracts and fractions at the KOR |
| **Supplementary Data** | Peptidomics analysis sheet (separate Excel file) |


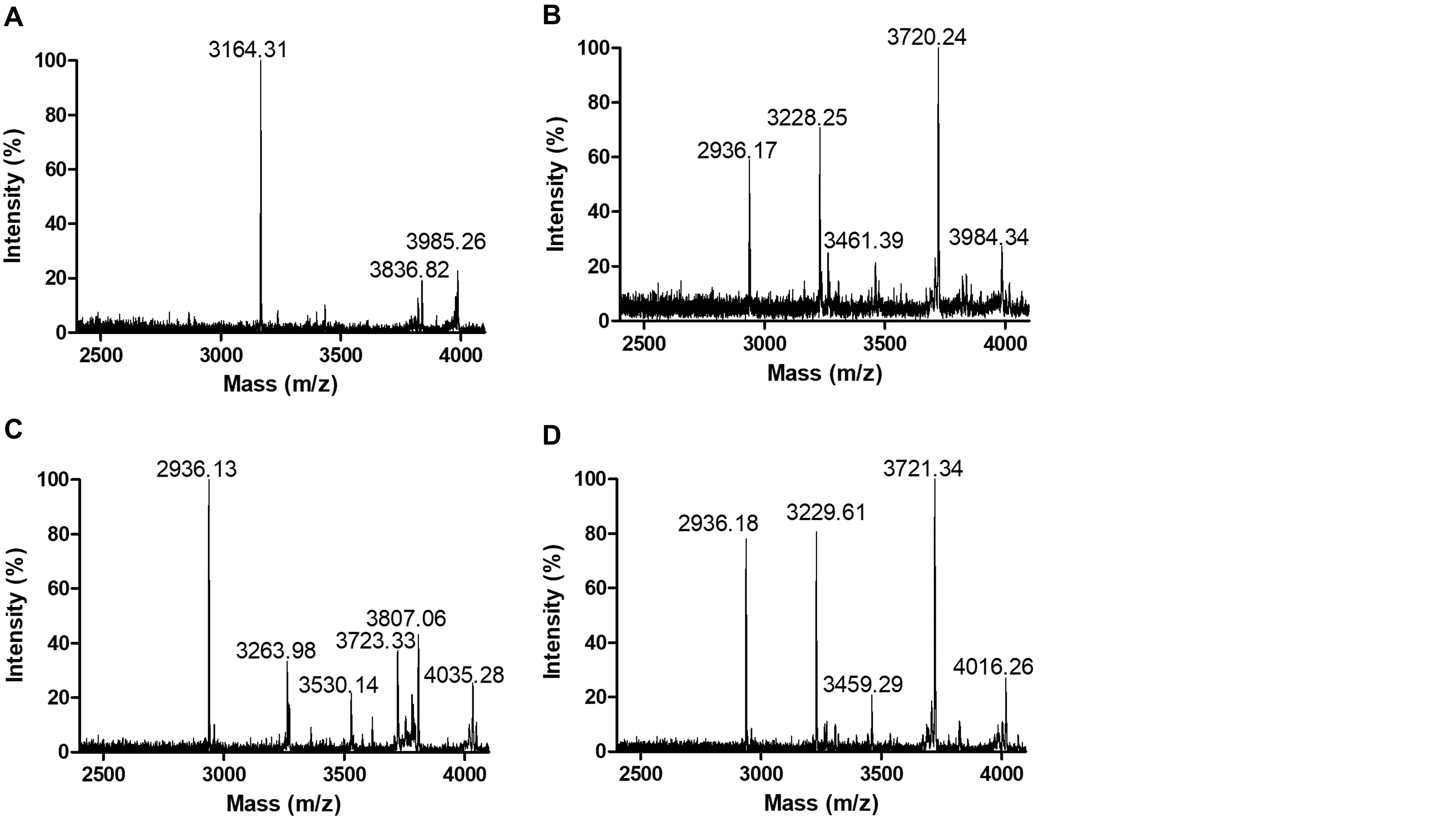


**Figure 1:** Representative MALDI-TOF MS chromatograms of scorpion venoms from five studied species. Panel (A) A. crassicauda, (B) M. eupeus, (C) H. saulcyi, (D) H. zagrosensis. Each chromatogram illustrates the molecular mass distribution of venom peptides, highlighting interspecific variations in the toxin profiles.


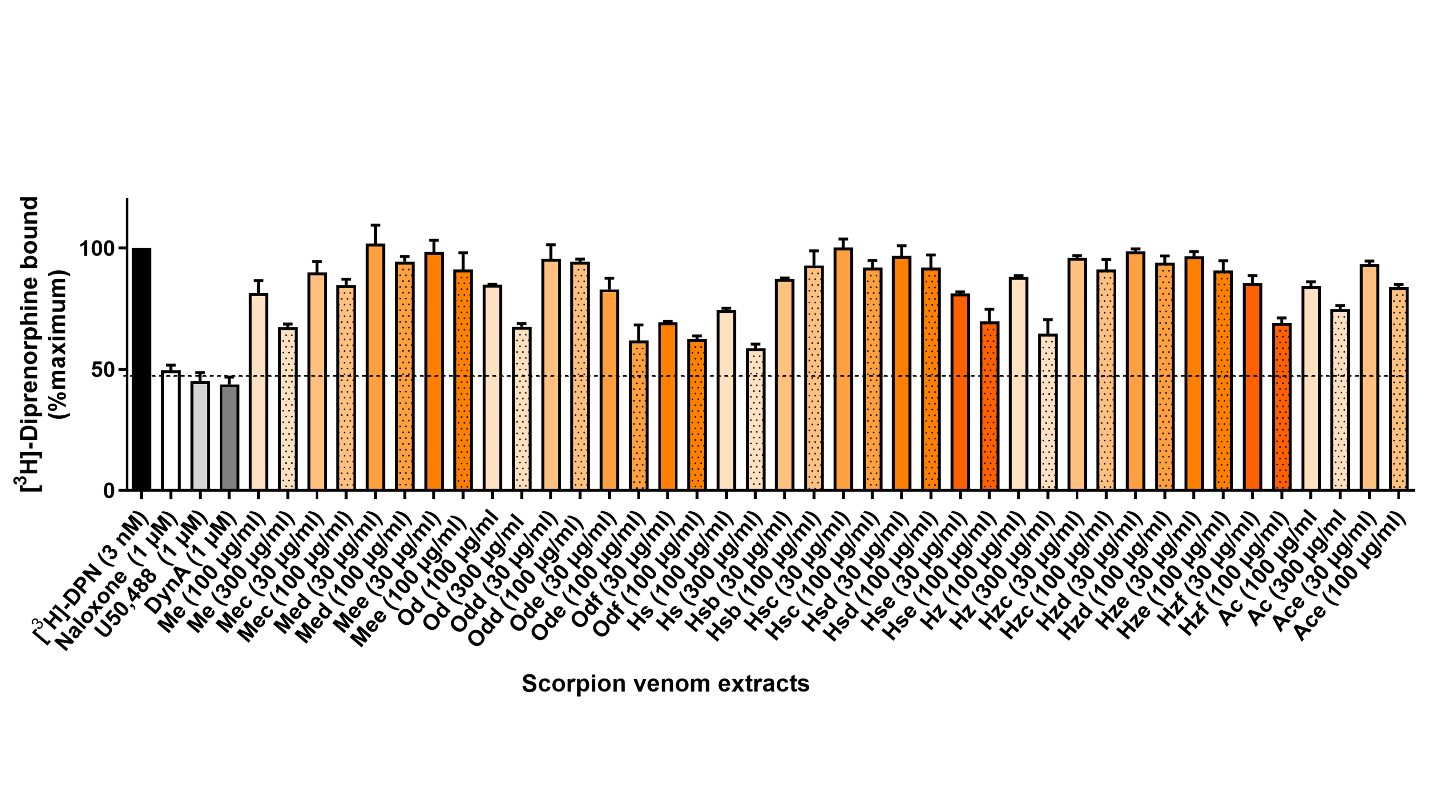


**Figure S2:** Radioligand displacement by crude venom extracts from Ac, Od, Me, Hs, and Hz to the KOR receptor (n=2), alongside a comparative analysis of SPE-purified venom fractions from the same species, highlighting the impact of purification on receptor-binding patterns (n=2). Here, the information and comparative analysis of the interactions of all fractions obtained from SPE in solvent B at elution percentages of 20% (b), 30% (c), 40% (e), and 50% (f) are presented. These results include both the crude venom and the SPE-eluted fractions of the scorpion species Ac, Od, Me, HS, and Hz.

**Table S1:** Molecular masses identified in crude scorpions’ venom by MALDI-TOF MS analysis^#^

| **Scorpion species** | **Name** | **Mass (m/z)** |
| --- | --- | --- |
|  | *A. crassicauda* | 3164.31, 3836.82, 3985.26 |
|  | *O. doriae* | 2949.24, 3168.42, 3651.56, 3704.55, 3794.00, 4057.34 |
|  | *M. eupeus* | 2936.12, 3228.26, 3461.40, 3720.25, 3984.35 |
|  | *H. saulcyi* | 2936.13, 3264.00, 3530.14, 3723.34, 3807.07, 4035.28 |
|  | *H. zagrosensis* | 2936.18, 3229.62, 3459.30, 3721.35, 4016.27 |

^#^comprehensive overview of the molecular masses identified in the crude venoms of five scorpion species using MALDI-TOF-MS analysis; this table systematically lists the species examined and presents the exact mass-to-charge (m/z) values of the ions detected for each species.

**Table S2:** Radioligand displacement at KOR by scorpion venom crude extracts and fractions

| **Sample** | **Mean normalized values [%]***^#^* | **±SD [%]** | **Sample** | **Mean normalized values [%]***^#^* | **±SD [%]** |
| --- | --- | --- | --- | --- | --- |
| [^3^H]-Diprenorphine  (3 nM) | 100 |  | Hs-b (30 µg/ml) | 87.4 | 0.5 |
| Naloxone (1 µM) | 49.6 | 4.8 | Hs-b (100 µg/ml) | 92.9 | 8.4 |
| U50 (1 µM) | 45.1 | 7.8 | Hs-c (30 µg/ml) | 100.4 | 5.0 |
| DynA (1 µM) | 43.9 | 6.8 | Hs-c (100 µg/ml) | 92.1 | 4.1 |
| Me (100 µg/ml) | 81.5 | 7.2 | Hs-d (30 µg/ml) | 96.9 | 5.9 |
| Me (300 µg/ml) | 67.4 | 1.8 | Hs-d (100 µg/ml) | 91.9 | 7.5 |
| Me-c (30 µg/ml) | 90.1 | 6.3 | Hs-e (30 µg/ml) | 81.4 | 1.0 |
| Me-c (100 µg/ml) | 84.9 | 3.3 | Hs-e (100 µg/ml) | 69.7 | 7.2 |
| Me-d (30 µg/ml) | 101.9 | 10.9 | Hz (100 µg/ml) | 88.2 | 0.8 |
| Me-d (100 µg/ml) | 94.5 | 3.0 | Hz (300 µg/ml) | 64.7 | 8.3 |
| Me-e (30 µg/ml) | 98.5 | 6.8 | Hz-c (30 µg/ml) | 96.0 | 1.4 |
| Me-e (100 µg/ml) | 91.3 | 9.7 | Hz-c (100 µg/ml) | 91.1 | 6.0 |
| Od (100 µg/ml) | 85.1 | 0.1 | Hz-d (30 µg/ml) | 98.6 | 1.6 |
| Od (300 µg/ml) | 67.6 | 1.8 | Hz-d (100 µg/ml) | 94.1 | 3.9 |
| Od-d (30 µg/ml) | 95.7 | 8.2 | Hz-e (30 µg/ml) | 96.8 | 2.7 |
| Od-d (100 µg/ml) | 94.5 | 1.6 | Hz-e (100 µg/ml) | 90.7 | 5.8 |
| Od-e (30 µg/ml) | 83.1 | 6.5 | Hz-f (30 µg/ml) | 85.5 | 4.6 |
| Od-e (100 µg/ml) | 61.9 | 9.1 | Hz-f (100 µg/ml) | 69.2 | 2.9 |
| Od-f (30 µg/ml) | 69.4 | 0.5 | Ac (100 µg/ml) | 84.3 | 2.6 |
| Od-f (100 µg/ml) | 62.6 | 1.8 | Ac (300 µg/ml) | 75.0 | 1.9 |
| Hs (100 µg/ml L) | 74.5 | 1.2 | Ac-e (30 µg/ml) | 93.6 | 1.7 |
| Hs (300 µg/ml) | 58.8 | 2.4 | Ac-e (100 µg/ml) | 83.9 | 1.6 |

^#^Measured counts per minute were normalized to the [^3^H]-Diprenorphine (100%); data is shown as mean values ± SD of bound radioligand; i.e. 100% correspond to lack of displacement of [^3^H]-diprenorphine.
